# Approach strategies and application of metabolomics to biotechnology in plants

**DOI:** 10.3389/fpls.2023.1192235

**Published:** 2023-08-11

**Authors:** Seon-Woo Oh, Muhammad Imran, Eun-Ha Kim, Soo-Yun Park, Sang-Gu Lee, Hyoun-Min Park, Jung-Won Jung, Tae-Hun Ryu

**Affiliations:** Division of Biosafety, National Institute of Agricultural Sciences, Rural Development Administration, Jeonju, Jeollabuk-do, Republic of Korea

**Keywords:** metabolomics, plants biotechnology, analytics, analytical instruments, safety assessment

## Abstract

Metabolomics refers to the technology for the comprehensive analysis of metabolites and low-molecular-weight compounds in a biological system, such as cells or tissues. Metabolites play an important role in biological phenomena through their direct involvement in the regulation of physiological mechanisms, such as maintaining cell homeostasis or signal transmission through protein–protein interactions. The current review aims provide a framework for how the integrated analysis of metabolites, their functional actions and inherent biological information can be used to understand biological phenomena related to the regulation of metabolites and how this information can be applied to safety assessments of crops created using biotechnology. Advancement in technology and analytical instrumentation have led new ways to examine the convergence between biology and chemistry, which has yielded a deeper understanding of complex biological phenomena. Metabolomics can be utilized and applied to safety assessments of biotechnology products through a systematic approach using metabolite-level data processing algorithms, statistical techniques, and database development. The integration of metabolomics data with sequencing data is a key step towards improving additional phenotypical evidence to elucidate the degree of environmental affects for variants found in genome associated with metabolic processes. Moreover, information analysis technology such as big data, machine learning, and IT investment must be introduced to establish a system for data extraction, selection, and metabolomic data analysis for the interpretation of biological implications of biotechnology innovations. This review outlines the integrity of metabolomics assessments in determining the consequences of genetic engineering and biotechnology in plants.

## 1 Introduction

Metabolomics refers to the technology for the comprehensive analysis of metabolites and low-molecular-weight compounds in a biological system such as cells or tissues (Pérez-Alonso et al., 2018). Metabolomics allows the systematic identification and quantification of these small molecules that are involved in biochemical interactions of organisms, thereby elucidating complex biological interactions, responses, and functions. The development and engineering of organisms through biotechnology (synthetic biology) exhibit new biological functions, as a functional expression of the genes involved is linked to metabolomes via transcriptomes and proteomes regulation (Moses et al., 2013). Similar to genomics, that refers to all the genetic information of an organism, the metabolome refers to the metabolic end products, which include hundreds to thousands of metabolites, depending on the scale of the organism or biological system involved. The qualitative or quantitative changes of metabolites in living organisms can be validated through various instrumental analyses and can be used to interpret various physiological phenomena or mechanisms of cells or organisms examination for comprehensive understanding of the complex physiology and biochemistry of organisms (Sharanya et al., 2020).

Metabolites are produced and modified by the metabolism of biological systems (such as cells, tissues, or organisms) such as peptides, amino acids, nucleic acids, lipids, carbohydrates, organic acids, vitamins, polyphenols, alkaloids, minerals, and chemical compounds absorbed and synthesized by cells or organisms. These compounds undergo changes in metabolic levels due to different environmental stimuli (biotic and abiotic), and by analyzing these environmental factors, the organism can be characterized, and metabolomic responses by specific factors can be predicted in connection with the mechanism of metabolic pathways (Poltronieri et al., 2013). Unlike microorganisms or animals, in plants more than 200,000 different metabolites have been reported (Weng et al., 2021). These metabolites constitute the substrates of enzymatic reactions and their qualitative and quantitative changes can determine the phenotype of organisms. Plant metabolomics aims to elucidate metabolic pathways and to allow the mathematical formulation of the total metabolic flux in terms of systems biology (Sharma et al., 2018). To find genes involved in the synthesis or breakdown of certain metabolites, quantitative trait loci (mQTL), and metabolic genome-wide association studies (mGWAS) in plant breeding, metabolome data can be integrated with genome and transcriptome data (Sakurai, 2022). Additionally, as the genomes of numerous plants have been sequenced, the demand for functional genomics approaches has encouraged the development of multi-omics-based strategies, with metabolomics playing an increasingly significant role. Thus, one of the most difficult fields is plant metabolomics, which has been shown to have an impact on crop improvement, the identification of bioactive substances, plant development, and responses to stress. Systems biology methods enable the integration of metabolomics with other -omics data, such as genomes, transcriptomics, and proteomics, to provide us a more comprehensive understanding of metabolic network control and cellular processes (Pinu et al., 2019).

The research on plant metabolites is of great value, and the design of experimental models is instrumental in deriving highly reliable results in metabolic profiling. When designing an experimental model, the selection and control of variables need to be carefully considered to ensure the integrity of the statistical analysis. Research on the safety assessment of crop composition needs to be divided into genetically modified organisms (GMO) and non-GMO groups, based on the independent variable of “genetic modification.” All other variables need to be held unchanged as control variables (Moghissi et al., 2018). After designing of the experimental model, the next step is to prepare high-quality samples. In the case of mass spectrometry (MS)-based metabolomics, the quality (such as homogeneity) of the samples is important. In order to ensure high quality and reliability in sample preparation, it is important to select samples representative of different environments (regions) of plant growth and the number of replicates needs to be considered for improved reliability in statistical analyses. In a study by Kim et al. (Kim et al., 2018) on the safety assessment of genetically engineered crops, a systematic approach was used for the preparation of crop samples and for analytical procedures. A crop composition database was developed using a diverse range of cultivars and regions so as to enhance the accuracy and reliability of the collected data.

There has been growing emphasis on the importance and necessity of metabolomics in terms of ‘big data’ utilization. Integrating big data with the metabolomic data and environmental information data with statistics, computer science, and bioinformatics can lead to a new approach for metabolite data validation. Therefore, big data-based safety assessment techniques need to be developed for organisms and plants engineered by biotechnology so that their values can be determined with verification of their safety. The nutritional value and safety of food and feed from GM crops is well informed by the quantitative, validated compositional methods for list of key analytes defined by crop-specific OECD consensus documents. Untargeted metabolic profiling has yet to provide data that better informs the safety assessment of GM crops than the already rigorous Codex-defined quantitative comparative assessment. Through the establishment of a systematic protocol with metabolite-level data processing algorithms, statistical techniques, and database development, metabolomics can be utilized and applied to products of biotechnology requiring safety assessment.

## 2 Metabolomics

Metabolites present in plants are low-molecular weight compounds such as lipophilic compounds in the plant cell wall, polar compounds from hydrophilic parts of the cell membrane, acidic and basic ions, and stable and oxidized structures. The metabolite levels change due to exogenous (environmental stimuli, such as drought, high temperature, circadian etc) or endogenous (homeostatic, diseases, growth and senescence) changes. This is because the metabolome (metabolites), the form of end products that determine a cell’s function, are linked to the proteome (protein products) and the genome containing genetic information of metabolites (Sharma et al., 2018). Therefore, in order to examine specific reactions in plants developed through mutations or engineered through biotechnology, integration of metabolomic information with genomic and proteomic information will be useful for deriving results with more significant implications and meanings. Additionally, it made it possible to gain a greater understanding of food composition, create new nutritional markers, and eventually build metabolic engineering techniques and the use of plants as bioreactor organisms. Plant metabolites are gaining interest in terms of human health due to their demonstrated protective benefits against diseases including diabetes, hypertension, and cancer, among others. But no one analytical platform or separation method can fully define the entire metabolome. However, calls for the use of metabolomics for compositional analysis of GM crops are being made due to the ongoing development of highly sensitive analytical tools and improved bioinformatic tools, in order to comparative assess more metabolites than those listed in the OECD consensus documents (Authority et al., 2018).

In plant metabolomics, a phased and systematic process from crop cultivation to analysis is needed in order to ensure meaningful interpretation of the results as well as reliability and accuracy of the results. In metabolomics, optimized detection of compounds can be achieved according to the extraction methods and the method of instrumental analysis used for identification and quantification of the compounds. Therefore, appropriate extraction solvents and analytical instruments need to be selected in consideration of the properties of the samples. For the implementation of efficient and systematic metabolomics, the necessary details of analyte extraction methods, instrumental analysis, data processing, and statistical analysis need to be considered and a combination of different extraction condition and instrumentation setting may be required to achieve broad coverage of metabolite classes.

### 2.1 Sample collection: field trial design

In order to perform metabolomics analysis for the safety assessment of a biotech crop, it is necessary to cultivate the biotech crop and a control comparator, as well as a reference of commercial varieties, in the same region to obtain compositional data under same environmental conditions. In addition, the test must be designed to have sufficient statistical power to identify significant differences between the biotech crop and the control crop (Benevenuto et al., 2022). To assess statistical natural variation in crop composition, environmental information (soil composition, climate, site, year of planting) and genetic background (varieties) should be collected so that correlation analyses can be performed as needed. EFSA recommended using at least four replicates throughout the entire study and cultivating at least three different crop varieties in at least eight sites for at least one year concurrently with a control group in order to conduct a comparative assessment of crop components (Ramos-Peralonso, 2014).

### 2.2 Sample preparation: storage and homogenization

The methods of grinding and storing the analytic samples have an impact on the recovery rate of the extracts. In general, a method of drying the moisture in a sample is used to minimize loss while preserving the properties of the constituents, which includes heating or freeze-drying. However, since drying by heating may alter the properties of the compound, freeze-drying is mostly used. Samples after drying can be stored in a freezer at -80°C or in liquid nitrogen. The methods of quenching and freeze-drying with liquid nitrogen are sometimes used to stop the metabolic process of organisms during the sample extraction process, (Mushtaq et al., 2014). The sample size needed for the analysis varies depending on the concentration of the target metabolites, pretreatment techniques, and analytical techniques, but it commonly falls between 10 and 100 mg fresh weight. Controls for consistent sampling in a short period of time are necessary for large-scale sampling, such as that required for mGWAS investigations, because the metabolome is sensitive to environmental influences (Gemmer et al., 2021; Yao et al., 2021). To prevent chemical changes caused by enzymatic and chemical reactions, a quenching method such liquid nitrogen freezing and extraction with organic solvents should be carried out right away.

Moreover, the homogenization of the sample is important for accurate quantification of the target extract. Use of a grinder, mill or a homogenizer can break down the matrix of the sample to increase extractability of small molecules and ensure homogeneity (Lin et al., 2007). If necessary, the extraction rate can be increased by using ultrasound-assisted extraction with the grinding method. In this approach, ultrasound is used to increase the solvent permeability while grinding to improve the extraction of the compounds. Ultrasound-assisted extraction is becoming a a common grinding method, and has been reported to be effective in extracting a variety of types of metabolites (Weng et al., 2021). Due to shortcomings in one or more study design elements, the results from these studies are challenging to interpret. These limitations include (1) not using validated and/or appropriately replicated test materials (e.g., samples from single, non-replicated growing conditions), (2) using test samples that are not relevant to the intended area of investigation (e.g., food/feed safety inquiries that examine non-consumed plant tissues), and/or (3) lacking data to characterize natural variability of the components that were analyzed. That additionally, the bialoaphos resistance gene (bar), which confers resistance to the broad-spectrum herbicide glufosinate, causes two plant endogenous metabolites, aminoadipate and tryptophan, to be acetylated in GM plants. (Christ et al., 2017).

### 2.3 Extraction methods and conditions

**Figure 1:**
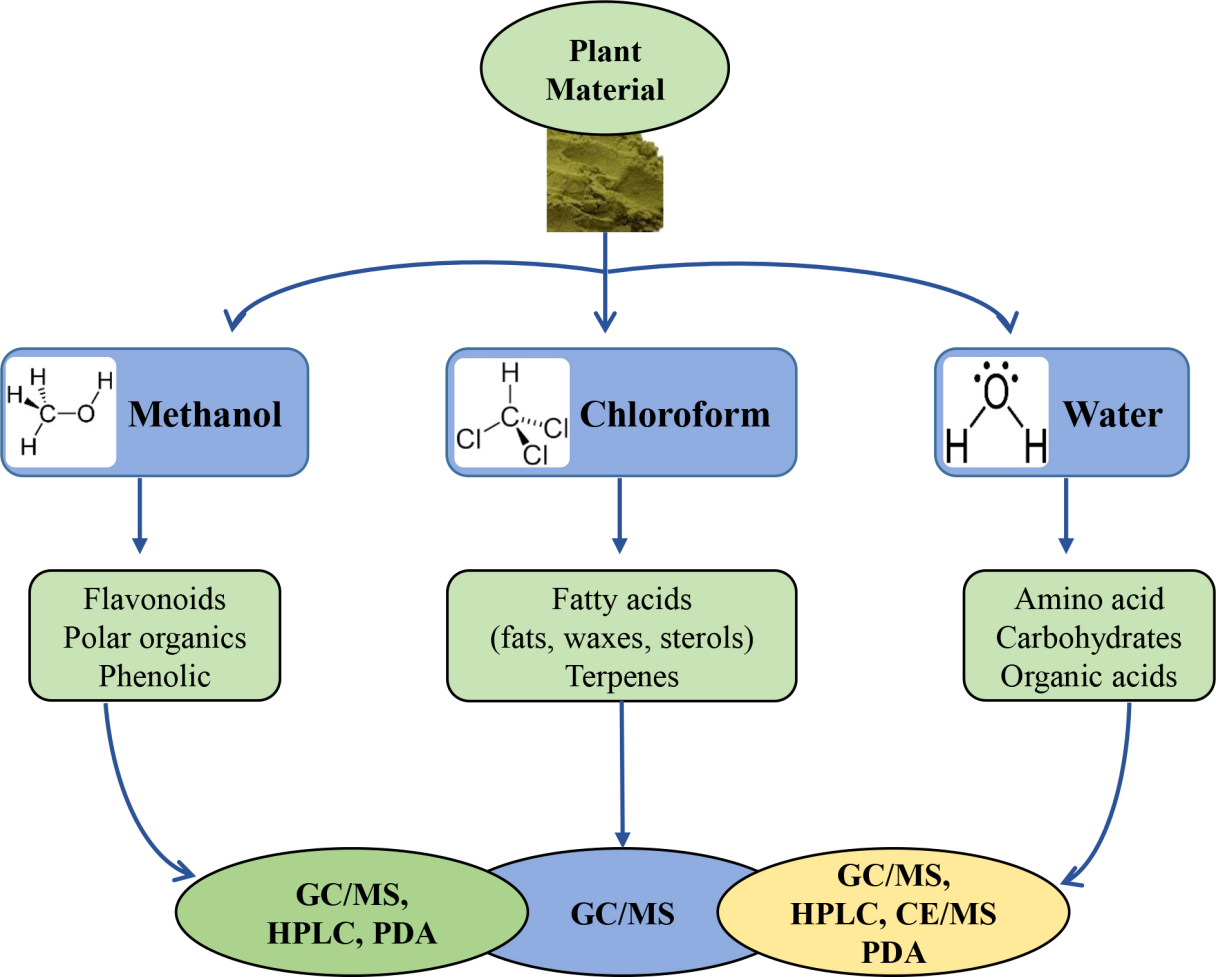
Selection of extraction solvents and instrumentation according to the properties of chemical compounds and analytical methods.

Solvent-based extraction of plant metabolites uses organic solvents with varying polarities, such as methanol, ethanol, and water for polar metabolites; and chloroform for lipophilic metabolites (**Figure 1**). The water solvent extracts salts, ions, carbohydrates, amino acids, and organic acids, and the extracted compounds can be analyzed using a variety of methods such as capillary electrophoresis (CE)–MS, high-performance liquid chromatography (HPLC)–photodiode array detector (PDA)–MS and gas chromatography (GC)–MS. In the case of methanol extraction, phenolic, flavonoid, saponin, and polar organic compounds can be extracted and can be analyzed using HPLC–PDA–MS or GC–MS. Lipids and terpenes extracted with chloroform can be analyzed using GC–MS (**Figure 1**). Depending on the characteristics of the analytical instrument used, the method of sample preprocessing for extraction may vary. In the case of analysis using GC–MS, there must be a derivatization process that converts non-volatile metabolites into volatile ones to improve the detection of compound by the GC-MS instrument. Attaching a trimethylsilyl (TMS) group to polar compounds containing functional groups such as OH, -COOH, -NH, and SH is a common derivatization to increase volatility. The reagents used for this method include N-methyl-N-(trimethylsilyl) trifluoroacetamide (MSTFA), Bis(trimethylsilyl) acetamide (BSA), and N, O-bis-(trimethylsilyl) trifluoroacetamide (BSTFA) (Zeki et al., 2020a), while MSTFA is most commonly used for plant metabolite profiling and quantification of sugars (Putri et al., 2022).

Perchloric acid extraction yields a protein-free extract after the denaturation of proteins, and is extensively used, owing to the advantage of immediately quenching enzymatic reactions. This extraction method is also commonly used for the extraction of polar, hydrophilic, or ionic metabolites from plant or animal tissues. Metabolites with different polarities can be extracted with mixtures of two or more solvents. For example, a solvent system of water/methanol/chloroform is used for the simultaneous extraction of polar and hydrophilic metabolites as well as non-polar metabolites (**Table 1**). These extraction methods with a mixture of solvents are widely used in analysis with high-performance analytical instruments such as liquid chromatography-tandem mass spectrometry, nuclear magnetic resonance (NMR) spectroscopy, and capillary electrophoresis (CE).

**Table 1:** Polarity and physical properties of extraction solvents.

Solvent	Polarity Index	BP (°C)	Solvent	PolarityIndex	BP (°C)
cyclohexane	0	80.7	ethyl acetate	4.3	77.1
Heptane	0		propanol, 1-	4.3	97.2
n-hexane	0	68.9	propanol, 2-	4.3	82.4-117.7
n-decane	0.3	174.1	methyl acetate	4.4	56.3
i-octane	0.4	99.2	cyclohexanone	4.5	155.7
octane	0.4	99.2	methyl ethyl ketone (MEK)	4.5	80
butyl ether	1.7	142.2	nitrobenzene	4.5	210.8
carbon tetrachloride	1.7	76.5	benzonitrile	4.6	191.1
triethyl amine	1.8	89.5	dioxane, 1,4-	4.8	101
i-propyl ether	2.2	68.3	dioxane, p	4.8	101.3
toluene	2.3	101.6	ethanol	5.2	78.3
xylene, p-	2.4	138	nitroethane	5.3	114
t-butyl methyl ether	2.9	55.2	pyridine	5.3	115.3
benzene	3	80.1	acetone	5.4	56.3
benzyl ether	3.3	288.3	benzyl alcohol	5.5	205.5
dichloromethane	3.4	40	methoxyethanol, 2-	5.7	124.6
methylene chloride	3.4	39.8	acetic acid	6.2	117.9
chloroform	3.4-4.4	61.2	acetonitrile	6.2	81.6
dichloroethane	3.7	83.4	dimethyl formamide, N,N-	6.4	153
ethylene dichloride	3.7	83.5	dimethyl sulfoxide	6.5	189
butanol, 1-	3.9	117.2	methanol	6.6	64.7
i-butyl alcohol	3.9	117.7	formamide	7.3	210.5
tetrahydrofuran	4.2	66	water	9	100

The Polarity index and physical properties such as boiling point (BP) of different solvent used for the simultaneous extraction of polar and hydrophilic metabolites as well as non-polar metabolites.

### 2.4 Instrumental analysis

For quantitative and qualitative profiling of plant metabolites, analytical separation techniques, such as chromatography are required for the detection of analytes within a given range of wavelength, mass, or chemical properties. In metabolomics, metabolite profiling is conducted using spectral peaks detected based on mass spectrometry. MS-based identification and quantification are techniques of analytical chemistry used for determining the type and quantity of a compound present in a sample by measuring the mass of a molecule (or fragments constituting the molecule) based on the mass-to-charge ratio (m/z) and the abundance of gas-phase ions. The mass spectrometer instrument provides an excellent analytical platform for metabolomics with its high sensitivity, reproducibility, and versatility (Zhao and Li, 2020). To perform MS, all samples need to be in gas phase, and the method of vaporizing the sample may vary depending on the state or properties of the sample. The metabolomic data can be obtained with maximized analytical throughput by direct injection in the GC–MS, LC–MS, and CE–MS. In most cases of metabolomic analysis, MS or NMR are used for quantification, and the most commonly used methods of MS are GC–MS, LC–MS, and CE–MS (Zeki et al., 2020; Wishart et al., 2022).

To achieve comprehensive profiling of the complete set of metabolites thereby increasing the qualitative detection performance for all classes of metabolites, two types of analytical instruments may be used in tandem to complement each type of instrument. For detection of secondary metabolites of plants, LC–MS is mainly used, since the instrument offers a broad spectral range for detection of a wide range of plant metabolites. GC–MS is considered to be more efficient for the detection of fatty acid-based compounds for which LC–MS shows limitations. GC–MS analysis is performed following a derivatization process, enabling analysis of non-volatile compounds, as well as high reproducibility and stability of data compared to that of HPLC. However, the chemical derivatization process may modify the chemical and structural properties of the raw material, and only the fragments formed by derivatization may be detected without detecting the other compounds that were not derivatized, resulting in limitations to achieving comprehensive profiling of the metabolites.

Analytical instruments for spectroscopy include NMR (Nuclear Magnetic Resonance) and FT–IR (Fourier transform infrared), which are used for metabolic profiling. The NMR is one of the significant tools for metabolite analysis directly from plant material, thereby minimized the loss sample and metabolite identification during the extraction process and the method also provides detailed information on the structure of metabolites; thus, the technique is used for identification of unknown compounds (Deborde et al., 2017). Of the common NMR techniques, ^1^H-NMR (proton NMR or hydrogen-1 NMR) is capable of compound identification and quantification a wild range of metabolites; the method has the advantage of simple and fast sample preprocessing, by oven dry the sample and then diluted with CD_3_OD and KH_2_PO_4_ buffer along with TMSP as an internal standard, however it also has limitations in low sensitivity and fewer number of detectable metabolites compared to GC–MS and LC–MS (Li et al., 2019b; Zeki et al., 2020b). Thus, 1H-NMR is mainly used for targeted metabolomics, which requires the quantification of specific metabolites (Riekeberg and Powers, 2017). Furthermore, using FT–ICR–MS (Fourier transform–ion cyclotron resonance–MS, also referred to as FT-MS) for metabolomics, offers a high resolution and mass accuracy, and is capable to provide simultaneous determination of hundreds to thousands of metabolites isotopologue species in a short time. The CE–MS instrument is based on a principle of separating compounds according to the differential migration of metabolite ions in an applied electric field, and it has the advantage in the analysis of hydrophilic ionic compounds and is useful for detecting phosphorylated compounds (Ren et al., 2018). However, it is difficult to achieve reproducibility of results since the operation of the instrument requires experience and skill.

## 3 Data collection and analysis

### 3.1 Data processing

Using the results derived from the instrumental analyses, structures are compared with reference standards to enable identification and quantification is performed based on comparison of peak areas. In the case of targeted metabolomics, the peak signals from the chromatography detected from the given wavelength range are compared with those of reference standards for each analytical instrument to perform identification and quantification. In the case of MS-based analysis, there may be cases of compounds that are not detected due to poor ionization because of interference or low sensitivity (Eicher et al., 2020). Therefore, to deal with the poor ionization a compatibility with analytical instruments should be taken into account when choosing solvents, to prevent metabolite degradation or alteration brought on by extraction conditions or enzymatic activities in tissue samples, sample preparation in SLE must be adjusted. Similarly, an SPE might be an effective way to extract the material and get rid of impediments (such salts or plentiful metabolites, for example). It has been found that a useful enrichment technique is to keep low abundant chemicals on the solid phase of SPE. In this regards, the three most popular chromatographic separation modes are normal phase, reverse phase, and ion-exchange are all covered by a variety of solid phase sorbents, including silica, alkylated silica (C-18), carbon-based sorbents, ion-exchange materials, polymer materials, and RAM (restricted access materials). Furthermore, the use of an internal standard allows correction for the loss of analyte during sample preprocessing. In order to improve the quality of the peak signals and reduce the bias present in the spectra, metabolomic data is subject to multiple preprocessing steps. Thereby, of spectra obtained from NMR and MS-based analysis, low-intensity peaks and noise peaks from the solvents or instruments used in the experiments are filtered using baseline correction; the optimal peak signal from only the sample analyte needs to be determined. Since it is believed that “omics” analysis provides a more thorough assessment of the GM variety for unintended changes than is possible by targeted analyses, it is suggested that a tiered approach could lessen the need for animal feeding trials with whole foods, which are currently required as part of the risk assessment process in Europe (Mesnage et al., 2016; Kok et al., 2019).

Peak overlapping occurs in the NMR or MS-based analyses, which can be resolved by spectral deconvolution. However, this method has limitations in untargeted metabolomics because we need to know the information of metabolites corresponding to the peak to be detected in the spectral range (Emwas et al., 2019; Wishart et al., 2022). Input conditions for data analysis can be set according to the analytical instrument and the method of spectrum processing used in the experiment. Of the types of metabolomics, targeted metabolomics allows measurement of primary changes caused by biotic/abiotic factors through quantification of a compound specific to the target trait in a biological system (Zhu et al., 2016; Weed, 2019). The spectral deconvolution method is suitable for direct quantification of the specific metabolite or related compound changed by external factors; detailed characterization of the molecular properties of the metabolite is performed. In targeted metabolomics, reliability of overall results is ensured by correction through normalization of measured values and maintaining data quality (Razzaq et al., 2019). Therefore, for analysis of a targeted metabolite, it is important to use a standardized method and, validated process of the analytical procedure to derive reproducible results. Profiling of targeted metabolites related to specific metabolic pathways can be performed by quantifying the metabolites that are changed by the mechanism of the related enzyme(s).

In the case of untargeted metabolomics, analytical instruments with high-performance/resolution are required for effective analysis. The advantages of the method include the analysis of the correlation between metabolites in a variety of metabolic pathways and the identification of unknown metabolites other than the targeted metabolites. However, the method of untargeted metabolomics is not able to obtain information on all metabolites due to various interference phenomena between compounds. This is because metabolites with no information in the search database and standard libraries such asNIST/EPA/NIH (NIST 14) mass spectral library, Fiehn_chemical (sequential), TMS_Chemical (sequential), Metline and in-house laboratory library (RDA) cannot be identified, but at the same time, the method has the advantage in that extensive metabolomic data can be obtained. Profiling of non-target metabolites is challenging due to the need for accurate mass values and comparisons to target metabolites, but can be addressed by upgrades in publicly available metabolome libraries and improved accuracy of chromatogram peak matching algorithms as equipment becomes more sophisticated. As GC MS is the most common analytical method and is suitable for the analysis of relatively small molecular weight, volatile and nonpolar substances, whereas LC MS, on the other hand, has a wider range of analyzable molecular weights and is effective for the analysis of highly polar substances. Therefore, it is possible to improve the detection of non-target metabolites by cross-using these two instruments.

### 3.2 Metabolomic profiling

**Figure 2:**
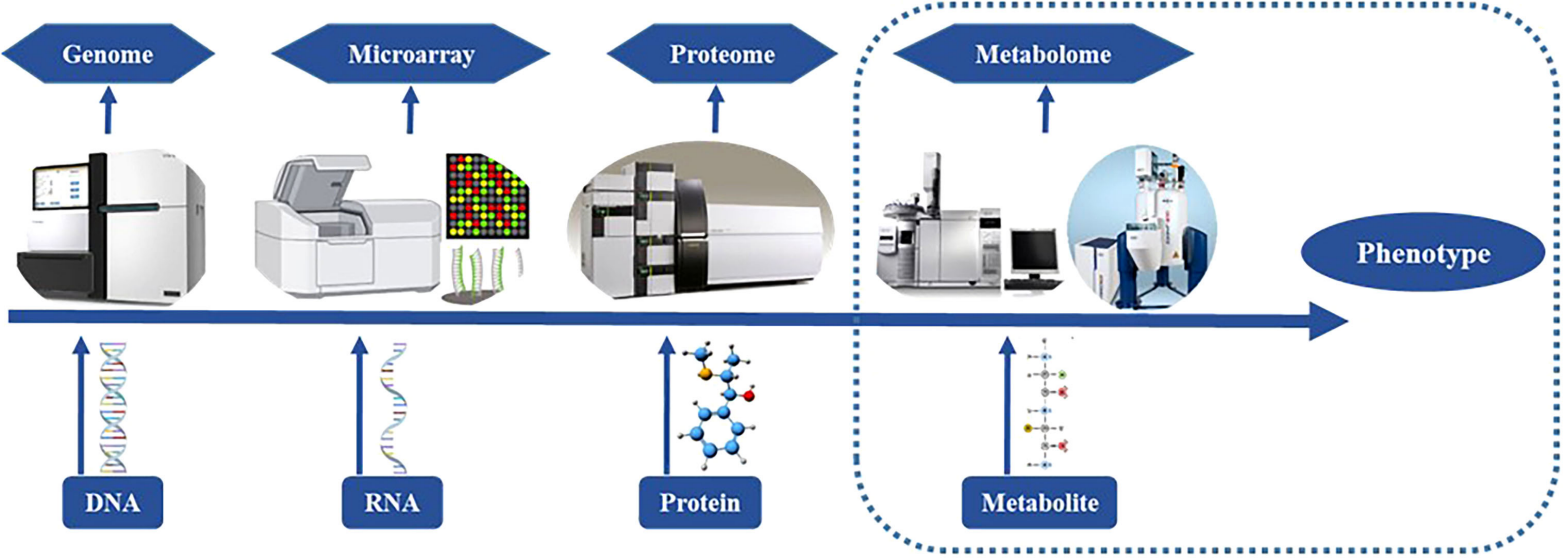
Assessment and utilization of different tools for Omics approach (genomics, microarray, proteome and metabolome).

Metabolites and functional compounds play a major role in biological metabolic processes, as such the integrated analysis of targeted and untargeted metabolites or network mapping for correlation analysis can signify the potential role of metabolites in different biological processes. Many studies have validated the significance of metabolites and their role in the plant development and their effects according to environment (**Table 2**). Metabolites have a range of functions, including those of fuel, structure, signaling, enzyme stimulation and inhibition, catalytic activity (usually as a cofactor), defense, and ingestion. These metabolites fall into one of two categories: primary metabolites, which include substances like lactate, carbohydrates, vitamins, and hormones, and secondary metabolites, which are produced biosynthetically and contain a variety of active substances with high biological activity (Bedair and Glenn, 2020). The four main chemical families that make up secondary metabolites are alkaloids, flavonoids, steroids, and pigments. The hallmarks of targeted metabolomics include high specificity, high detection sensitivity, and reliable quantitative data obtained through high throughput instrumental analysis. It can reveal the relevant molecular mechanisms and offer strong support for the ongoing study, development, and use of metabolic molecular markers through the quantitative and qualitative analysis of target metabolites in plant tissues in combination with other experimental data (Cardoso et al., 2019). When novel qualities of crops are generated, metabolomics information can play a role in giving quick and crucial information regarding unintentional differences since it is most directly related to the phenotypic characteristics of plants (**Figure 2**). Non-targeted metabolomics is a method for investigating vast amounts of metabolites and revealing properties and interactions in an organism and highlights the substances that have accumulated during a certain growth stage in an organism. A highly helpful technique for discovering unique changes in an organism at the screening level is non-targeted analysis. Complex metabolic matrices in biotech plants can offer a quick way to compare and assess the output (Perez De Souza et al., 2019). Because of this, metabolomics research requires the fusion and interpretation of vast volumes of data from a range of devices, the most popular of which are nuclear magnetic resonance and mass spectrometry (Scossa et al., 2016). As these procedures may be important in the detection of metabolites that play critical role in plant and human nutrition and defense response, such as carbohydrates serve as molecules for energy storage and transport (starch) and structural molecules (cellulose, hemicellulose and lignin) in biological systems (Nelson and Cox, 2005; Wingard et al., 2014). Monosaccharides are the basic units of carbohydrates and include galactose, fructose, and glucose. Multiple monosaccharides are linked together to form polysaccharides (Niaz et al., 2020) (**Table 3**). Organic acids can be converted into monosaccharides (such as glucose) and are synthesized into polysaccharides (such as starch). Pyruvate, lactic acid, glycerol, 3-phosphoglyceric acid, and amino acids are involved in gluconeogenesis. Thus, gluconeogenesis and glycolysis are regulated separately. Furthermore, polysaccharides and glycans are synthesized by dedicated glycosyltransferases. In this mechanism, uridine diphosphate glucose (UDP-glucose), a nucleotide sugar, is sequentially added to the hydroxyl group of the growing polysaccharide chain for polymerization. The synthesized polysaccharides may serve structural or metabolic functions as they are, or may be, linked to lipids or proteins by oligosaccharyltransferases (Kay et al., 2019; Sim et al., 2022). Those the lipids and proteins serves as the most concentrated source of energy, and constitute the bilayers of biological membranes (Nelson and Cox, 2005). The acyl chain of the fatty acid is elongated by a cycle of reactions that add the acetyl group, which reduce it to an alcohol, and dehydrate it to an alkene group, and then reduce it again to an alkane group (Mullen and Yet, 2015; Wase et al., 2017) (**Table 4**). In similar, the proteins act as enzymes and catalyze chemical reactions in metabolism. Nucleotides are made from amino acids, carbon dioxide, and formic acid. The synthesis of purine nucleotides is an edge case use of amino acids whose canonical roles include protein synthesis, synthesis of chemical signals and mediators, and catabolism for energy (Davies et al., 2011; Gautam and Chahota, 2022). Adenine and guanine are synthesized from inosine monophosphate (IMP), a nucleoside precursor, and pyrimidines are synthesized in the form of heterocyclic oxide of glutamine (Gupta et al., 2021) (**Table 5**). As a result, all proteins and eventually metabolites that interact with a certain protein of interest and affect its activity or expression are included in the protein’s interactome. The development of a protein interactome may provide details on the purpose of the protein and all of the molecular actions in which it takes part and protein interactomes in disease may also reveal dysfunctional pathways, how they are controlled, and probable protein partner involvement in the illness (Martins-De-Souza, 2014). Similar to this, the phosphatidylinositol 3-kinase and mammalian target of rapamycin pathway (PI3K-mTOR) is crucial for treating MDD because it results in inflammatory cytokines activating immune cells. A 33 components of the PI3K-mTOR pathway have been studied extensively utilizing the Y2H screen as part of an interactome analysis. More than 800 interactions, including 67 unique ones, are present in the PBK-mTOR pathway (Weichhart and Säemann, 2008). A number of MS techniques, including Q, QqQ, IT, or TOF, have been utilized, depending on the level of sensitivity, mass resolution, and range required. For example, MALDI-assisted TOF/MS is suitable for precise and quantitative single-cell metabolite profiling, low mass protein detection (with a mass range of 1-300 kDa), and low mass protein detection with a high sensitivity of around 10-18 nanomolar (Marchev et al., 2020). Moreover, the most critical step for the successful application of these metabolites in the market is to profile them with accuracy in different sources such as plant-based sources (fruits, vegetables, leaves, seeds, etc.) is require susing various sophisticated methods and techniques in order to boost their detection and use at a quicker rate for the development of novel medicines, nutraceuticals, chemical discovery, food safety, and quality.

**Table 2:** Current studies use different tools for metabolite profiling under different environmental condition in conventional or GM crop varieties.

Analytical Tool	Test Study	Crop	Detected Metabolites	References
LC-MS/MS; MALDI–MSI	Salinity	*Hordeum vulgare*	PC, fatty acyls, SQDAG, glycerolipid, prenol lipid, polyketide, DAG, and sphingolipid	(Sarabia et al., 2018)
LC-TQMS	Drought	*Medicago sativa; M. truncatula*	carbohydrate, Flavonoid, proline and abscisic acid	(Echeverria et al., 2021)
GC-MS	Salt, cold,	*Physcomitrella patens*	organic acid, Sugar, and amino acid	(Arif et al., 2018)
GC-TOF MS; LC-MS/MS	High light, cold	*A. thaliana*	Sugar, hexose, amino acid, gluconic acid, organic acid, Citrate,	(Küstner et al., 2019)
FIE-HRMS,	Nutritional improvement	Peral Millet	dietary starch, antioxidants and vitamins	(Yadav et al., 2021)
LC-MS/MS	Pathogen resistance	Maize	benzoxazinoid and Flavonoid,	(Zhou et al., 2019)
LC-MS	Salt tolerance	Maize	amino acids, Terpenoids, lipids, benzoxazinoids, and flavonoids	(Liang et al., 2021)
LC-MS	Seed oil-related traits	Soybean	arginine, aspartic acid, asparagine, Alanine, and daidzein	(Liu et al., 2020)
LC/UPLC-MS/MS; GC-MS	Drought	*A. thaliana*	TAG, DGDG, SQDG, PC, MGDG, PS, PE, and PI	(Salem et al., 2020)
GC-MS; LC	Drought	*Oryza sativa*	Aconitic acids, benzoic acid, Citric, carbohydrates, norvaline, proline, GABA, benzoic acid,	(Auler et al., 2021)
LC-ESI-MS/MS	Environment adaptation	Foxtail Millet	Phenolamides, lipids, hydroxycinnamoyl derivatives, and flavonoids	(Wei et al., 2021)
LC-MS/MS	Flavonoid pathways	Wheat	Flavonoids	(Chen et al., 2020)
LC-MS/MS	Grains, plant height	Wheat	deoxyinosine-5′-monophosphate and Betaine,	(Shi et al., 2020)
ESI-QqTOF-MS/MS	Fruit traits	Tomato	Polyphenol, vitamins, polyamine, alkaloids, and Amino acid	(Zhu et al., 2018)
GC-TQMS	Salinity	*Oryza sativa*	organic acid, Mannitol, and sugar,	(Chang et al., 2019)
LC-ESI-MS	Fruit quality	Strawberry	anthocyanins, flavonoids, and Phenolics,	(Labadie et al., 2020)
LC-MS/MS	Agronomic traits	Rice	feruloylserotonin and L-asparagine	(Li et al., 2019a)

**Table 3:** Metabolite profiling of carbohydrates.

Category		Carbohydrates
**General structure**		Aldoses, Ketoses, Furanose, Pyranose, Glycosidic bond
**Geometrical structure**		Anomers, Epimers, Mutarotation
Monosaccharides	Trioses	Aldotrioses (Glyceraldehyde), Ketotrioses (Dihydroxyacetone)
	Tetroses	Aldotetroses(erythrose, threose), Ketotetroses(erythrulose)
	Pentoses	Aldopentoses(arabinose, lyxose, ribose, xylose) Ketopentoses(ribulose, xylulose), Deoxy sugars (deoxyribose)
	Hexoses	Aldohexoses(allose, altrose, galactose, glucose, mannose), Ketohexoses(fructose, tagatose), Deoxy sugars (fucose, fuculose, rhamnose)
	Heptoses	Ketoheptoses(mannoheptulose, sedoheptulose)
	Nonoses	Neuraminic acid, Sialic acid
**Disaccharides**		Cellobiose, Isomaltose, Isomaltulose, Lactose, Lactulose, Maltose, Sucrose, Trehalose, Turanose
Polysaccharides	Trisaccharides	Maltotriose, Melezitose, Raffinose
	Tetrasaccharides	Acarbose, Stachyose
	Oligosaccharides	Fructo-oligosaccharides-FOS, Galactooligosaccharides -GOS, Isomalto-oligosaccharides -IMO, Maltodextrin, Mannan oligosaccharides (MOS)
	Other polysaccharides (Complex carbohydrates)	Beta-glucans (Oat beta-glucan, lentinan, sizofiran, zymosan, cellulose, chitin), Chitosan, Dextrin, Dextran, Fructan (Inulin), Galactan, Glucan, Hemicellulose, Levan, Lignin, Mannan, Pectin, Starch(Amylopectin, Amylose), Xanthan gum

Carbohydrates profiling, the first column expresses two general categories such as geometrical structural carbohydrates and disaccharides carbohydrates, while the second column expresses the carbohydrates present their respective category.

**Table 4:** Metabolite profiling of lipids.

Category	Lipids
**General classification**	Saturated fat, Unsaturated fat, Monounsaturated fat, Polyunsaturated fat
**Geometrical classification**	Trans fat, Omega-3, Omega-6, Omega-7, Omega-9
**Eicosanoids**	Arachidonic acid, Prostaglandin, Prostacyclin, Thromboxane, Leukotriene
**Fatty acids**	Caprylic acid, capric acid, lauric acid, myristic acid, palmitic acid, stearic acid, arachidic acid, behenic acid, lignoceric acid
**Glycerides**	Monoglyceride, Diglyceride, Triglyceride (Triheptanoin, Trimyristin, Tripalmitin, Tristearin, Trilinolein, Triolein)
**Phospholipids**	Phosphatidylserine, phosphatidylinositol, phosphatidylethanolamine, cardiolipin, dipalmitoylphosphatidylcholine
**Sphingolipids**	Ceramides
**Steroids**	Cholesterol, Corticosteroids, Sex hormones, Secosteroids

The first column shows the different classes of lipids such as general class, geometrical, Eicosanoids, Fatty acids, Glycerides, Phospholipids, Sphingolipids, and Steroids. while the second column shows the lipids present in their respective class.

**Table 5:** Metabolite profiling of proteins.

Category		Proteins
Properties	Aliphatic	Glycine, Alanine, Proline, Methionine, Valine, Isoleucine, Leucine
	Aromatic	Phenylalanine, Tyrosine, Tryptophan
	Polar uncharged	Serine, Threonine, Cysteine, Asparagine, Glutamine
	Positive charged	Lysine, Arginine, Histidine
	Negative charged	Aspartic acid, Glutamic acid
Type	Essential amino acids	Lysine, Threonine, Leucine, Isoleucine, Valine, Methionine, Phenylalanine, Tryptophan
	Conditional amino acids	Glutamine, Glycine, Cysteine, Arginine, Serine, Tyrosine, Proline
	Positive charged	Arginine, Histidine, Lysine
	Negative charged	Aspartic acid, Glutamic acid
	Polar uncharged side chains	Serine, Threonine, Asparagine, Glutamine
	Special cases	Cysteine, Selenocysteine, Glycine, Proline
	Hydrophobic side chain	Alanine, Isoleucine, Leucine, Methionine, Phenylalanine, Tryptophan, Tyrosine, Valine
	Glucogenic	The 20 standard amino acids excluding leucine and lysine
	Ketogenic	Leucine, Lysine + Tryptophan, Tyrosine, Threonine, Isoleucine, Phenylalanine
	Secondary amino acids	Proline(the only proteinogenic secondary amino acids), Azetidine-2-carboxylic acid, Pipecolic acid, Hydroxyproline (non-proteinogenic cyclic secondary amino acids), Sarcosine (acyclic secondary amino acids)

Category of different proteins on the base of their properties and types and the proteins present in their respective category.

#### 3.2.1 Metabolite profiling of plant-derived functional compounds

Terpenes and isoprenoids are lipids containing carotenoids and are the largest group of plant natural products (Kim et al., 2016). The compounds are synthesized through polymerization and transformation of isoprene units from the reactive precursors, isopentenyl pyrophosphate (IPP) and dimethylallyl pyrophosphate (DMAPP). In plants, IPP is synthesized using pyruvic acid and glyceraldehyde 3-phosphate. Isoprene donors are involved in the steroid biosynthesis pathway. In the steroid biosynthesis pathway, isoprene units are combined into squalene, which is cyclized into lanosterol. Lanosterol is a tetracyclic triterpenoid and is involved in the biosynthesis of steroids such as lanosterol which converted into other steroids, like cholesterol and ergosterol in animals and fungi, where plant steroids are synthesized through cycloartenol. (Jäpelt and Jakobsen, 2013; Lee et al., 2020). In this regard, the simultaneous characterisation and dereplication of active substances in complex mixtures, such as extracts of different plant spices, is made possible by affinity selection-mass spectrometry (AS-MS), UHPLC-MS size-based separation techniques, NMR, GC-MS etc (Muchiri and Van Breemen, 2021). Those the metabolites in plants are expected to be simultaneously measured by metabolic profiling. Metabolic profiling can be done using a number of analytical methods, including (GC-MS), (LC-MS), and (NMR) (Muchiri and Van Breemen, 2021). Numerous hundred compounds from a variety of classes, such as sugar, organic acids, amino acids, alcohols, amines, and fatty acids, can be identified using GC-MS. Similarly, Pyroline-5-carboxylate synthetase (P5CS), which converts glutamate to pyrroline-5-carboxylate (P5C), is an enzyme that metabolizes both proline and glutamate. Pyroline-5-carboxylate reductases (P5CR) finally produce this stress-responsive amino acid from the reduction of P5C (Meena et al., 2019). Considering the nutritional qualities of fruits in the human diet, the antioxidant potential is among the most nutritious rich crops having multi-minerals, proteins, essential amino acids, carbohydrates, vitamins, fatty acids, phytosterols, carotenoids, polyphenolics and flavonoids as reactive oxygen species. Similarly, crops containing secondary metabolites quercetin 3,7-di-O-α-l-rhamnopyranoside and narigenin-7-O-β-d-(3-p-coumaroyl)-glucopyranoside have desirable nutritional benefits. The high content of carbohydrates, organic acids (ascorbic, citric, malic, quinic, succinic, fumaric and oxalic acids), capsaicinoids (capsaicin, dihydrocapsaicin, nordihydrocapsaicin, homocapsaicin, homodihydrocapsaicin), carotenoids, flavonoids, luteolin and vitamins (E, C and A) that add to the antimicrobial, antiseptic, anticancer, counterirritant, appetite stimulator, immunomodulator and antioxidant properties (Batiha et al., 2020). The metabolite profiling of plants based secondary metabolites have been considering as most vital component in nutritional and safety assessment by utilizing UHPLC-Q-TOF-MS, GC-MS and LC-MS based metabolomics assessment in different plants species.

### 3.3 Statistical analysis and visualization in metabolomics

Data may be referred to as a set of variables and observations collected by selecting parameters in line with the purpose of analysis and designing an appropriate data collection and sampling method to obtain the desired information. Therefore, sufficient information on the data collection method (location and time) and sampling method needs to be presented to enable proper data analysis. As the results of metabolomics may differ depending on the normalization of the data, the process of normalization for the analyzed raw data is imperative. For example, if the distribution of the data is non-normal, or if there are large differences in the magnitude of the values due to different units for the variables, or if there are missing values, we can normalize the data using logarithmic transformations, square-ratio transformations, min-max normalization, etc. to improve the accuracy of the analysis results. In order to examine the overall trend of data distribution, a box plot or histogram may be used to grasp the trend in the overall data at a glance. The utilization of statistical techniques in metabolomics is highly important in -omics research. In particular, in multivariate analysis, the number of metabolomic variables is reduced to a small number of new variables, so that the high-dimensional data matrix can be reduced to lower dimension matrix with new axes, thereby enabling the examination of the overall data patterns (Barnes et al., 2016). Because the purpose of multivariate analysis is to make use of the correlation between variables rather than the analysis of causal relationships, it is characterized by the absence of dependent variables. In the case of data with a causal relationship with the dependent variable, the correlation analysis can be performed through regression analysis or ANOVA (Eicher et al., 2020).

**Figure 3:**
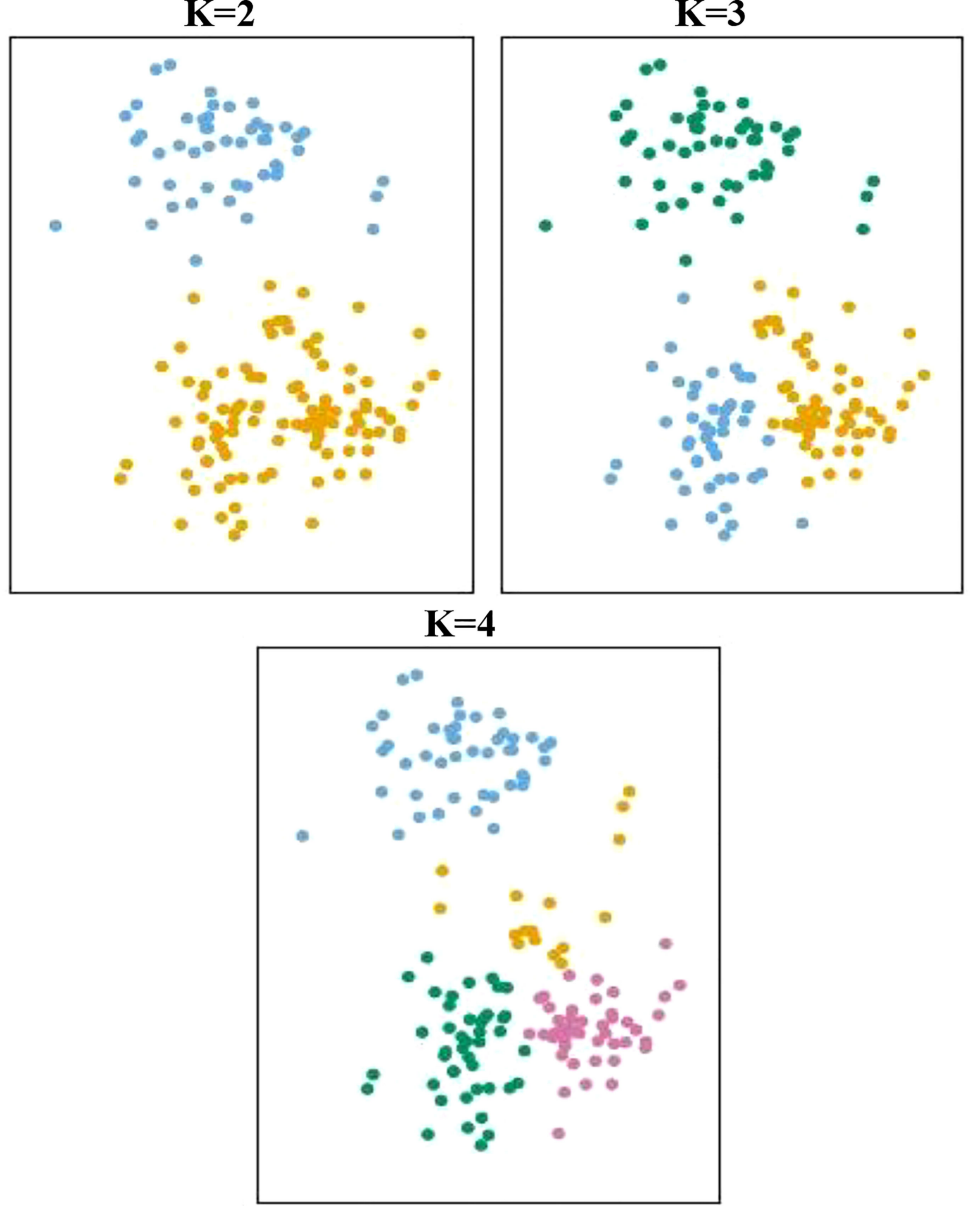
**K** principal components and clustering on a two-dimensional plane.

Multivariate analysis includes Principal Component Analysis (PCA), Factor Analysis (FA), Cluster Analysis (CA), and Discriminant Analysis (DA) (Chen et al., 2018). PCA is mainly used when the purpose is to reduce dimensionality due to a large number of variables (as in the case with metabolomics). Principal Component Analysis (PCA) is used for sorting data into an order or classification, and is also used as a method of resolving a problem called multicollinearity in regression analysis (Mafata et al., 2022). PCA is a method of reducing the dimensionality of the original variables by obtaining k (<*n*) independent principal components from *n* variables, formed as a linear combination (Chatfield, 2018) (**Figure 3**). PCA is an unsupervised learning method for effectively identifying data patterns for the data to represent biological changes. The method is based on one-dimensional transformation to uncorrelated (orthogonal) variables (known as principal components) of multidimensional metabolomics data. Other analysis methods based on unsupervised learning include hierarchical clustering analysis and the self-organizing map. The difficulty in determining whether the observed changes among the thousands of signals described by the untargeted profiling method(s) have any bearing on safety is one major barrier to employing data from omics research with GM crops, including metabolomics. A suggestion to quantify omic comparisons using a one-class SIMCA (Soft Independent Modelling of Class Analogies) model was published in 2014 (Van Dijk et al., 2014). Transcriptomic data from six commercial potato cultivars with a track record of safe use were used to construct a multivariate one-class classification classification model.

**Figure 4:**
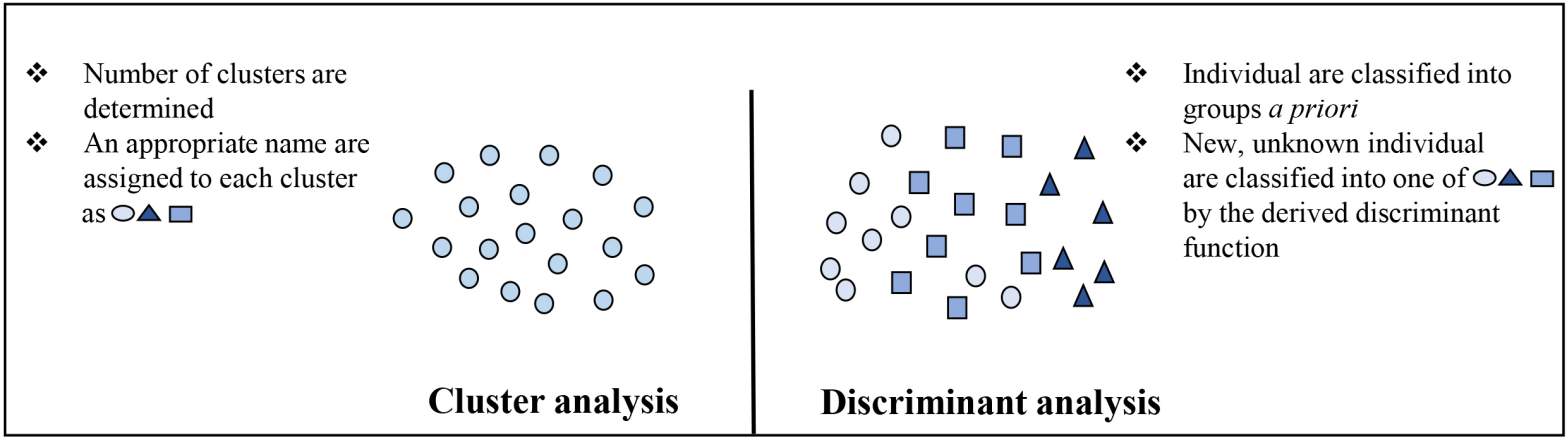
Comparison of cluster and discriminant analysis.

The purpose of factor analysis (FA) is to determine the relationship between *n* variables and divide them into *m* (<*n*) groups of variables. Examining the group of variables formed in FA, the coefficient of correlation of within-group variables is high and the coefficient of correlation of between-group variables is low (Zhao et al., 2021). PCA explains the variability in the variables by expressing them into orthogonal linear combinations of the original variables, whereas FA expresses the original variables as an orthogonal linear combination and explores the relationship between the variables (Lever et al., 2017). Moreover, the cluster analysis (CA) method of grouping target individuals into clusters with similar features in consideration of various features of the individuals and visualizing the clusters in a low-dimensional space. Measured variables are used to obtain distances between individuals (similarity) to perform classification. In CA, it is not known to which cluster the individuals are assigned before analysis; Multi-Dimensional Scaling (MDS) is an example. Cluster analysis enables visualized classification, but similarity must be calculated as values to actually perform classification of individuals by similarity. Discriminant analysis (DA) also known as canonical discriminant analysis, is a multivariate technique used to separate two or more groups of observations (individuals) based on variables measured on each experimental unit (sample) and find the contribution of each variable in separating the groups DA is a statistical technique that enables determination of the group to which a target individual belongs, based on a discriminant function when groups (two or more) are known *a priori* (**Figure 4**).

In order to overcome the limitations of the PCA method when reducing the dimensionality using linear relationships, a partial least squares (PLS) method is introduced, which reduces dimensionality and increases the predictive performance by deriving latent variables that consider the correlations with the dependent variables. Since the PLS–DA method, which combines PLS and Linear Discriminant Analysis (LDA) methods, was proposed (Lasalvia et al., 2022), it has been used in a wide range of applications. OPLS is a method in which orthogonal component is added to PLS, and when there is only one response variable (Y), Orthogonal Partial Least Squares–Discriminant Analysis (OPLS–DA) is used. The OPLS–DA method is used to determine the difference between two groups (Thevenot, 2016). By rotating the data matrix so that the difference between the observed groups appears in the first singular vector, the difference between the observed values and the groups can be easily identified. Since there are many analysis variables in metabolomics, use of PLS–DA and OPLS–DA methods, which are combined with discriminant models, allows more clear identification of the classification patterns (Thevenot, 2016).

## 4 Metabolomics and genomics in plants

Similar to the terms genomics, transcriptomics, and proteomics, “metabolomics” refers to the study of a live organism’s metabolome and encompasses metabolite identification, measurement, and interactions. The metabolite profile of an organism is altered by changes to genes and proteins (Gomez-Casati et al., 2013). Similar to other omics, high throughput metabolite screening techniques are available. For example, a combination of gas chromatography and mass spectrometry (GC-MS) was used to compare the metabolic profiles of different genotypes to show that the metabolic phenotypes diverged from each other more than they did from each mutant from its parent ecotype, indicating that the cell metabolome is influenced more by the difference in ecotype than by a single mutation (Zhang et al., 2022). The results of metabolomics must be evaluated to connect anomalies in metabolites to potentially inadequate reactions/enzymes and their related genes in order to integrate metabolomics and genomics. For this, a few strategies have already been created such as IEM is employed for metabolic networks (Baumgartner et al., 2004; Bongaerts et al., 2022).

Similarly, transgenic potato plants expressing specific genes were subjected to pair-wise metabolite-to-metabolite and transcript-to-metabolite correlation analyses, which revealed novel connections not previously proposed by conventional targeted techniques (Oksman-Caldentey and Saito, 2005; Zhang et al., 2022). A large percentage of these transgenic plants showed no discernible characteristics. There were no obvious phenotypic changes in the Arabidopsis pal1 and pal2 mutants lacking the activity of the two phenylalanine ammonia lyase genes. The particular function of PAL1 and PAL2 was proposed by the phenotyping of the single mutants and the double-mutant using a combination of transcript profiling and in-depth targeted metabolite profiling for sugars, amino acids, and phenylpropanoids (Vanholme et al., 2010). For Arabidopsis lines overexpressing the PAP1 gene, which codes for a Myb-like transcription factor, integrated transcriptome analyses and a thorough chemical analysis using LC-MS and FT-ICR MS were performed (Zhang et al., 2022). The enhanced accumulation of anthocyanins was the only factor contributing to the modifications in metabolic profiles brought on by PAP1 gene expression. The expression of genes known to be involved in the manufacture of anthocyanins was elevated, and as a result, other upregulated genes, such as members of the glycosyltransferase, acyltransferase, and glutathione S-transferase families, might be tentatively ascribed a function in the generation of anthocyanins. Experimental testing using T-DNA-inserted knockout mutant lines and *in vitro* enzymatic tests using recombinant proteins validated the function of some of these putative genes (Chorfi et al., 2022; Yin et al., 2022). These methods show that integrating transcriptome and metabolomics analysis in functional genomics research on Arabidopsis is feasible (Yin et al., 2022). The re-programming of the transcriptome and metabolome is modulated by nutritional and abiotic stressors. As a result, an integrated analysis identifies the gene functions that these stresses modulate. A study on sulfur starvation in Arabidopsis provided a nice illustration of this (Oksman-Caldentey and Saito, 2005; Zhao and Rhee, 2022).

When metabolomics is combined with disease GWASs, the examination of disease molecular mechanisms improves from an understanding of the genetic mechanisms behind the variation in metabolite levels. Metabolite levels are biological readouts of age, environment, lifestyle, and the genome. Numerous metabolite levels have thousands of genetic correlations (metabQTLs) identified by GWAS (Hollywood et al., 2006; Kastenmüller et al., 2015). similarly, Sun and co-workers highlighted the huge potential for crop development in connecting certain metabolites and metabolic pathways linked to health and nutrition. and concentrate on the use of metabolomics in rice, maize, soybean, wheat, and other crops and validate the effects of environment on the evaluation of the quality and metabolite variation of plant-derived products and food safety (Sun et al., 2020). In this regard, the application of metabolomics in breeding programs is led by metabolite quantitative trait loci (mQTLs) and metabolome-based genome-wide association study (mGWAS). Many metabQTLs’ underlying processes, however, are unknown. The majority of metabQTLs are found in non-coding genomic areas, but it is still unclear how much of this is due to changes in gene expression. MetabQTLs and transcriptomics data have been combined, although only for metabolite-specific transcriptomics data. Similar to this, utilizing the Metabolon mass spectrometry instrument, (Yin et al., 2022) examined plasma metabolites in 6,136 Finnish men from the Metabolic Syndrome in Men (METSIM) research and conducted GWASs for 1,391 metabolites, discovering 2,030 unique metabolite genetic correlations (metabQTLs).can improve our comprehension of the molecular causes of disease. These studies show that integrating transcriptomic and metabolomics results with GWASs individually complements the identification of genes underlying GWAS associations and that doing so reveals regulatory mechanisms underlying metabQTLs. This emphasizes the fact that integrating transcriptomic and metabolomics results together can help us better understand the molecular mechanisms underlying disease.

## 5 Utilization of metabolomics data for safety assessment of biotechnology crops

In the safety assessment of crops developed or engineered using biotechnology, changes in the components other than the protein expressed for the intended target trait are common factor to compared. In this case, the items of analysis are nutrients recommended in the OECD Biosafety Consensus Documents (Safety Assessment of Transgenic Organisms in the Environment), which correspond to target compounds of the safety assessment. The assessment method is based on the concept of substantial equivalence (Kearns, 2019). An -omics analytical approach that measures non-target compounds can be used depending on the target component of the analytes and its need. If comparative evaluation based on metabolomics, phenotypes, and agronomic characteristics is used, systematic assessment of unintended changes will be possible while expanding the concept of substantial equivalence.

Metabolomics of crops forms the basis for the overall understanding of the components using a range of metabolomics profiling, and is the basis of the monitoring the process. Through metabolomics profiling or the approach of targeted metabolites, metabolic synthesis pathways of natural components constituting the crop and enzyme actions can be understood. Because the traits of existing commercially approved genetically modified (GM) crops have clear metabolic pathways (such as herbicide and disease resistance) and are not metabolites of complex networks, it is possible to perform risk assessment with targeted metabolomics. In the case of methods not used previously or organisms introduced with new traits with no prior precedence, assessment is possible with current regulatory scientific techniques, but there is a need to develop new assessment techniques to reflect these changes. In the case of engineered crops that are similar in genetic variation to that found in traditional breeding (Bhandari et al., 2017), it will be difficult to perform safety assessments using the existing assessment methods. In this case, various methods, including the -omics technique, should be applied; the new crop should be compared with existing crops, new assessment methods should be proposed (Bhandari et al., 2017; Valle et al., 2019).

The -omics research allows more effective investigation of changes and causes of biological phenomena as a result of the comprehensive analysis of key compounds related to biological phenomena (such as genes, RNA, proteins, and metabolites expressed in cells or organisms), rather than individual analysis of each element. Research using metabolomics data includes a number of studies that analyze the effects of the environmental stress on plants, including: metabolic profiling analysis of the effect of salt stress on changes in the metabolite patterns of tomato using FT–IR (Skolik et al., 2019), analysis on the effects of cold-temperature stress (Davey et al., 2009) and herbicide stress on plant metabolites using high-performance mass spectrophotometry (Sulpice et al., 2009). These studies built metabolomics data and performed comparative analyses. Another study on the effect of environmental stress on plants investigated the stress tolerance of a plant using microarray and network analysis (Kim et al., 2016). In safety assessments of biotech crops using new technology, there has been research examining the synthesis of toxic substances to analyze unintended changes. For example, in crops with non-browning traits, comprehensive safety assessments can be performed by combining targeted or untargeted metabolomics data centered on metabolites such as polyphenols or glycoalkaloids. Using plant metabolomics, integrated with the analysis of the effects of biological and environmental factors, to analyze and compare metabolic mechanisms will pave the way for more valuable results.

## 6 Conclusion

Metabolites are small molecules produced during metabolism, play a vital role in biological phenomena through their direct involvement in the regulation of physiological mechanisms, such as maintaining cell homeostasis as well as signal transmission through protein–protein interactions. Understanding biological phenomena based on the integrated analysis of metabolomics mechanisms and biological information is crucial. Plant metabolomics research focuses on researching and comprehending all of a plant’s secondary metabolites from a comprehensive viewpoint, including the genome, transcriptome, and expressionome, in an omics-based approach rather than on isolating and identifying a single component of a plant. To learn more about metabolites from GC-MS, LC-MS, NMR, etc., several techniques are frequently used in conjunction in plant metabolomics research. Techniques for statistical data analysis are also required in order to mine the enormous amount of data that was collected. Additionally, significant developments in metabolomics and its incorporation into other omics (genomics, transcriptomics, and proteomics) have advanced our understanding of the connections between many levels of biological systems, paving the way for the realization of systems biology. Combining knowledge from the fields of biology, chemistry, instrumentation, and bioinformatics has made this possible. To determine all of the metabolites in a plant extract, metabolomics is primarily necessary. However, unlike DNA sequencers for genomics or DNA arrays for transcriptomics, there is no single technology for metabolomics, and it may never be practicable. This is due to the fact that a variety of chemistries are required for the study of metabolites with different physicochemical properties; unlike nucleic acids and proteins, metabolomics cannot be handled by a single chemistry. In terms of whole-genome sequencing, metabolomics is analogous to attempting it without either the Sanger method or the Maxam-Gilbert method. Currently, combinations of various high sensitivity analytical methods are typically employed for comprehensive nontargeted chemical analysis.

Recently, with the growing scope and application of research using bioinformatics, and their use in metabolomics has also been considerably increasing. In metabolomics, metabolites can be characterized by profiling and obtaining information on targeted and untargeted metabolites using high-performance analytical instruments such as MS in order to examine interrelationships in metabolic pathways.

Thereby, metabolomics profiling provides useful information on various physiological phenomena of organisms. In order to determine the extent of secondary, unintended changes in compounds (in addition to intentionally modified compounds in biological systems) as a result of bioengineering (such as genetic recombination), safety assessments can be done based on targeted and untargeted metabolomics, which includes the targeted and untargeted metabolomics compounds. Therefore, in safety assessments based on metabolomics, it is important to understand how the involved genes or substances are associated with the metabolic pathways and what consequences are predicted.

In order to achieve this goal, it will be necessary to develop comprehensive metabolomic databases and create corresponding maps of metabolite profiling. It is important to note that currently available equipment and technology might have limited information on metabolomics. As a result, drawing conclusive insights from the metabolic pathway maps could also be restricted. In order to overcome these limitations, integration of -omics data information for analysis is imperative. The information of metabolites obtained from a range of analytical instruments must be collected, classified, and extracted according to the purpose, and to this end, the introduction of information analysis technology will be required. Since this process requires considerable time and effort from data collection to analysis, it will be necessary to establish a system for a series of processes from data extraction, selection, and metabolomics data analysis for interpretation of biological implications of the findings.

## Author contributions

Conceptualization, methodology, resources and writing-original draft preparation and writing S-WO, MI and E-HK. Data collection S-YP, S-GL, and H-MP. Review and editing MI. Data curation J-WJ. Supervision T-HR. All authors contributed to the article and approved the submitted version.
